# Zero-shot stance detection: Paradigms and challenges

**DOI:** 10.3389/frai.2022.1070429

**Published:** 2023-01-13

**Authors:** Emily Allaway, Kathleen McKeown

**Affiliations:** Department of Computer Science, Columbia University, New York, NY, United States

**Keywords:** stance detection, zero-shot, multilingual, transfer learning, domain adaptation

## Abstract

A major challenge in stance detection is the large (potentially infinite) and diverse set of stance topics. Collecting data for such a set is unrealistic due to both the expense of annotation and the continuous creation of new real-world topics (e.g., a new politician runs for office). Furthermore, stancetaking occurs in a wide range of languages and genres (e.g., Twitter, news articles). While zero-shot stance detection in English, where evaluation is on topics not seen during training, has received increasing attention, we argue that this attention should be expanded to multilingual and multi-genre settings. We discuss two paradigms for English zero-shot stance detection evaluation, as well as recent work in this area. We then discuss recent work on multilingual and multi-genre stance detection, which has focused primarily on non-zero-shot settings. We argue that this work should be expanded to multilingual and multi-genre zero-shot stance detection and propose best practices to systematize and stimulate future work in this direction. While domain adaptation techniques are well-suited for work in these settings, we argue that increased care should be taken to improve model explainability and to conduct robust evaluations, considering not only empirical generalization ability but also the understanding of complex language and inferences.

## 1. Introduction

One major challenge for stance detection is the large (potentially infinite) and diverse set of stance topics in the real world. Furthermore, as more people around the world turn to online platforms for news or sharing opinions (e.g., Tweeting, commenting on news articles), stance models must be able to generalize to new topics regardless of language or genre. However, due to both the expense of annotation and the continuous creation of new topics (e.g., a new politician runs for office), it is unrealistic to collect data for all possible topics, languages, and genres. Therefore, ***zero-shot stance detection*** (**ZSSD**) (i.e., stance detection requiring zero-shot learning) is crucial for developing widely applicable stance models.

Studies in ZSSD typically do zero-shot learning over topics (i.e., evaluate on topics not seen during training; **ZSSD**^*Topic*^). Although this is similar to ***cross-topic stance detection***^1^, cross-topic stance requires human knowledge about the evaluation topics, since they are assumed to be semantically related to the training topics, making cross-topic stance less viable for broad generalization. Stimulated by our development of the VAST dataset (Allaway and McKeown, 2020) and our adaptation of existing cross-topic stance data to ZSSD^*Topic*^ (Allaway et al., 2021), English ZSSD^*Topic*^ has received increasing attention (i.e., Liu et al., 2021; Zhu et al., 2022) and follows two evaluation paradigms, namely, (i) many-topic (i.e., many unseen topics but very few examples per topic), and (ii) few-topic (i.e., only a few topics but many examples per topic). We survey existing work on these two datasets and argue that incorporating external knowledge (e.g., from Wikipedia) and training topic-invariant features are the most promising directions for further exploration.

Despite recent advances in English ZSSD^*Topic*^, most existing work on stance detection is single-domain. That is, models are trained and evaluated on texts that share some attribute (Plank, 2016), such as language, genre, and topic. Additionally, stance detection datasets include a variety of label sets (e.g., pro/con/neutral vs. agree/disagree/discuss/unrelated). We argue that in order for stance systems to become broadly applicable, ZSSD should be extended to include zero-shot learning across languages (**ZSSD**^***Language***^), genres (**ZSSD**^*Genre*^), and label sets (**ZSSD**^*LabelSet*^).

Although recent work on **multi-domain stance** (e.g., Schiller et al., 2021; Hardalov et al., 2022), explores transfer learning, it does not evaluate in specific ZSSD^*^ settings, where *∈{*Topic, Language, Genre, LabelSet*}. Therefore, in order to support and standardize further efforts in ZSSD, we propose a set of best practices. Additionally, we discuss the necessity in ZSSD of **evaluating models for robustness** (e.g., performance on challenging phenomena such as sarcasm, resilience to adversarial attacks) and **improving explainability**.

## 2. Paradigms for ZSSD

We provide an overview of ZSSD^*Topic*^ in English by describing the most common datasets (Section 2.1), as well as existing work on these datasets (Section 2.2) and its strengths and weaknesses (Section 2.3).

### 2.1. Data

We describe two datasets for ZSSD^*Topic*^: VAST (Section 2.1.1)—a dataset for many-topic stance covering a broad range of topics (Allaway and McKeown, 2020), and Sem16 (Section 2.1.2)—a Twitter dataset covering six topics (Mohammad et al., 2016) which has been adapted for few-topic stance. Examples of the two datasets are shown in Table 1. Although other datasets have been used for cross-topic stance (e.g., Conforti et al., 2020; Zhang et al., 2020), we discuss Sem16 because it is the primary dataset used for few-topic stance. In both datasets, the task is to predict a stance label ŷ∈{pro, con, neutral} for each input.

**Table 1:** Dataset statistics for VAST and Sem16.

		**# Exs**	**# Topics**		**Example**		
			**ZS**	**All**	**Text**	**Topic**	**Label**
VAST	*Train* *Dev* *Test*	13,477 2,062 3,006	0 383 600	4,641 497 759	There is only a shortage of agricultural labor at current wages. Raise the wage to a fair one, and legal workers will do it. If US agriculture is unsustainable without abusive labor practices, should we continue to prop up those practices?	agricultural labor wages	Con
Sem16^⋆^	*Train* *Dev* *Test*	3,542 621 707	0 0 1	5 5 0	Donald Trump is Biff in the alternate universe 1985 in Back to the Future 2. #biff #BackToTheFuture #SemST	Donald Trump	Con

We also include a single example from each dataset in the right portion of the table. ^⋆^Note that for Sem16, the number of examples depends on which topic is treated as the zero-shot test topic. We present the statistics for DT (Donald Trump) as the test topic. See Allaway et al. (2021) for detailed statistics on the number of examples for individual topics.

#### 2.1.1. VAST

The VAST dataset consists of comments from *The New York Times* with 5634 topics covering broad themes, such as politics (e.g., “a Palestinian state”), education (e.g., “charter schools”), and public health (e.g., “childhood vaccination”) (Allaway and McKeown, 2020). The topics also include a wide range of similar expressions (e.g., “guns on campus” and “firearms on campus”), capturing variation in how humans might realistically describe the same topic. Note that VAST has both zero-shot and few-shot evaluation sets.

#### 2.1.2. Sem16

The Sem16 dataset was created for SemEval2016 Task 6 and consists of English Tweets on six topics (e.g., “Donald Trump”) (see Table 1). Although the dataset was not developed for ZSSD, we adapted it for the few-topic setting by treating each topic *t* in turn as the zero-shot test topic (Allaway et al., 2021). Specifically, for each *t* all examples from the other five topics are used for training and validation and all examples from *t* are used for testing. This setting is different from cross-topic stance, where only two *related* topics are used, one for training and one for evaluation (e.g., Xu et al., 2018; Wei and Mao, 2019; Zhang et al., 2020). The zero-shot setup allows evaluation on a topic even when a related one is not provided and makes a larger dataset available for training each model.

### 2.2. Methods

Existing work on ZSSD^*Topic*^ uses a combination of techniques: (1) learning latent topics in order to generalize (Section 2.2.1), (2) leveraging pragmatic information (Section 2.2.2), (3) learning topic-invariant features (Section 2.2.3), and (4) incorporating external knowledge (Section 2.2.4). We discuss each of these in turn.

#### 2.2.1. Latent topics

For ZSSD^*Topic*^, especially many-topic, the large and diverse set of topics can be challenging for models. In order to exploit this variation, in our prior work (Allaway and McKeown, 2020) we assume a set of *K* latent topics underlies the larger set. For example, “*vaccinating children”* and “*flu shots”* would be part of a latent topic on “*vaccination.”* The latent topics provide implicitly-learned information about unseen topics during evaluation. While we proposed *fixed* latent-topic representations derived by clustering the input representations, following studies update latent-topic representations during training, either by reclustering (Liang et al., 2022b) or from randomly initialized embeddings (Liu R. et al., 2022). Although latent topics are less used for few-topic stance, since the number of topics is already small, they have been used for cross-topic stance to model the similarity between the training and test topics (e.g., Wei and Mao, 2019).

#### 2.2.2. Pragmatic information

Learning to identify which parts of a document (e.g., words and phrases) are most important for conveying the stance on a topic can help models identify general stance patterns and so generalize to new topics. Model attention, whether unguided (Xu et al., 2018) or directly trained to mimic human relevance scores (Jayaram and Allaway, 2021), encourages the model to learn this information. Additionally, explicit measures of word-usage, including relative frequency in a topic (Liang et al., 2021) and topic-specific keywords (Liang et al., 2022a) derived from LDA (Blei et al., 2003), can help models automatically identify important words for conveying stance.

#### 2.2.3. Topic-Invariant features

ZSSD^*Topic*^ can be framed as a domain-adaptation problem (i.e., the topic is the domain) and domain-invariant features are a common component of many domain adaptation techniques (e.g., Ben-David et al., 2006; Glorot et al., 2011; Ganin et al., 2016; Zhang et al., 2017). The aim of such features is to learn information that can generalize across domains. In stance detection, topic-invariant features (i.e., the topic as the domain) are obtained primarily through two techniques: contrastive learning (Baly et al., 2020; Liang et al., 2022b; Liu R. et al., 2022; Liu Y. et al., 2022) and adversarial learning (Wei and Mao, 2019; Baly et al., 2020; Allaway et al., 2021; Hardalov et al., 2021).

The aim of **contrastive learning** (e.g., Hadsell et al., 2006) is make the representation of an input (the *anchor*) similar to *positive examples* and different from *negative examples*. In stance detection, contrastive learning is applied to inputs with different topics to encourage topic-invariant feature representations. Often, stance labels (e.g., pro, con) are used to choose positive examples (Liu R. et al., 2022), but other properties have been used as well, including whether examples are topic-agnostic (i.e., their stance prediction doesn't depend on topic-related words) (Liang et al., 2022a) and whether two examples share a latent topic (Liang et al., 2022b). Negative examples are often the remainder of the anchor's mini-batch, but they can also be chosen to have a specific relation to the anchor (e.g., a different media source) (Baly et al., 2020; Liu Y. et al., 2022). In both many-topic or few-topic stance, contrastive learning encourages the model to focus on specific properties that hold across topics in order to generalize.

Similarly, **adversarial learning** (Ganin et al., 2016) directly forces the model to learn domain-invariant features by requiring that the same set of features is both useful for stance prediction and *not* useful for predicting the domain of an input. In order to learn topic-invariant features, the topic is typically treated as the domain (Wei and Mao, 2019; Allaway et al., 2021), although other domains have also been used such as the media outlet (Baly et al., 2020). Although adversarial learning with topic as the domain is well suited to few-topic stance (e.g., Allaway et al., 2021), it does not generalize broadly to many-topic stance, since the number of labels for the domain-classifier grows drastically, making training difficult.

#### 2.2.4. External knowledge

External knowledge is often drawn explicitly from an external source (e.g., Wikipedia articles related to a topic He et al., 2022; Zhu et al., 2022, commonsense knowledge bases Liu et al., 2021, sentiment and emotion lexica Zhang et al., 2020) and then used either as graphs (Zhang et al., 2020; Liu et al., 2021) or as raw-text that is passed with the input to a language model encoder (He et al., 2022; Zhu et al., 2022). Alternatively, knowledge can be incorporated indirectly through task pre-training (e.g., on ideology prediction Baly et al., 2020; Liu Y. et al., 2022). Regardless, this technique can generalize to a continually expanding set of topics (i.e., real-world settings), as long as relevant external knowledge is available, and can be applied to both few-topic and many-topic settings.

### 2.3. Discussion

We now present and discuss the results of the above methods for ZSSD^*Topic*^ (Table 2), both in the many-topic (on VAST) and few-topic settings (on Sem16). We note that we include cross-topic stance models in our results in order to present a full picture of progress. However, in our prior work (Allaway et al., 2021) we argued that the standard cross-topic assumptions about the similarity of training and test topics may impact the generalization ability of models. Therefore, we focus our analysis specifically on the zero-shot models.

**Table 2:** ZSSD results for many-topic and few-topic stance.

		**VAST (macro-*****F***1**)**		**Sem16 (** ***F***_*****avg*****_ **)**					
		**ZS**	**All**	**FM**	**LA**	**HC**	**DT**	**CC**	**A**
Baseline	BERT	0.660^‡^	0.653^‡^						
	BERT-finetune	0.685^†^	0.684^†^	0.419^♦^	0.448^♦^	0.496^♦^	0.401^♦^	0.373^♦^	0.552 ^♦^
LTop	TGA-Net	0.666	0.665	0.466^♣^	0.453^♣^	0.487^♣^	0.415^♣^	0.354^♣^	0.542^♣^
LTop, TInv	DTCL	0.708	0.712						
	JointCL	0.723	-	0.538	0.495	0.548	0.505	**0.397**	0.545
	^♢^VTN			0.478	0.473	0.364	0.477	-	-
Prag, TInv	PT-HCL	0.716	-	**0.546**	0.509	0.545	0.501	0.389	**0.565**
Prag	^♢^Cross-Net	0.434^‡^	0.455^‡^	0.431^♦^	0.442^♦^	0.418^♦^	0.461^♦^	-	-
	prior-bin:gold	0.693	0.684						
	^♢^TPDG			0.541	**0.583**	0.529	0.504	-	-
TInv	TOAD	0.410^♣^	-	0.541	0.462	0.512	0.495	0.309	0.461
TInv, EK	POLITICS	-	**0.767**						
	Baly	-	0.756 ^♠^						
EK	^♢^SEKT	0.418^†^	0.411^†^	0.513	0.536	0.420	0.477	-	-
	CKE-Net	0.702	0.701						
	TarBk-BERT	0.736	-	0.538	0.487	**0.551**	**0.508**	0.395	0.562
	WS-BERT-Single	**0.753**	0.745						

Best results are **bold**, second and third best are underlined. ^♢^ indicates a cross-topic model for few-topic stance.

^♢^

The topics for Sem16 are: FM (feminist movement), LA (legalization of abortion), HC (Hillary Clinton), DT (Donald Trump), CC (climate change is a real concern), A (atheism). *F*_*avg*_ is the average of pro and con classes only. Cross-topic topic pairs are: FM↔LA (i.e., train on FM and test on LA, and vise versa) and HC↔DT. CC and A are not used in cross-topic stance detection because they have no related topic for training.

Models are: TGA-Net (Allaway and McKeown, 2020), DTCL (Liu R. et al., 2022), JointCL (Liang et al., 2022b), VTN (Wei and Mao, 2019), PT-HCL (Liang et al., 2022a), Cross-Net (Zhu et al., 2022), prior-bin:gold (Jayaram and Allaway, 2021), TPDG (Liang et al., 2021), TOAD (Allaway et al., 2021), POLITICS (Liu Y. et al., 2022), Baly (Baly et al., 2020), SEKT (Zhang et al., 2020), CKE-Net (Liu et al., 2021), TarBk-BERT (Zhu et al., 2022), and WS-BERT-Single (He et al., 2022).

Models are split by techniques used: LTop, Latent Topics (Section 2.2.1); Prag, Pragmatics Information (Section 2.2.2); TInv, Topic-Invariant Features (Section 2.2.3); EK, External Knowledge (Section 2.2.4).

Results are taken from the cited papers except for: ^†^is from Liu et al. (2021), ^‡^is from Allaway and McKeown (2020), ^♠^is from Liu Y. et al. (2022), ^♦^is from Allaway et al. (2021), and ^♣^is from Zhu et al. (2022).

For zero-shot many-topic stance on VAST, including external knowledge is the most successful technique (Table 2). Interestingly, incorporating knowledge from Wikipedia (He et al., 2022; Zhu et al., 2022) is substantially better than incorporating commonsense knowledge (Zhang et al., 2020; Liu et al., 2021). Models adding external knowledge through task pre-training (Baly et al., 2020; Liu Y. et al., 2022) also perform well, achieving the **best performance on all topics**, including non-zero-shot ones (i.e., *All* *F*1). Since zero-shot topics make up 79% of the test topics (Table 1), zero-shot *F*1 is likely similar to the reported *All* *F*1. Although early models used latent topics (Allaway and McKeown, 2020) and pragmatics (Xu et al., 2018; Jayaram and Allaway, 2021), the addition of topic-invariant features has further improved performance (Liang et al., 2022a,b; Liu R. et al., 2022). In fact, combining topic-invariant features with other techniques (e.g., in the best models Baly et al., 2020; Liu Y. et al., 2022) outperforms using only the topic-invariant feature technique (Allaway et al., 2021).

Methods using external knowledge (Zhu et al., 2022) and contrastive learning (Liang et al., 2022b) are also successful in few-topic stance on Sem16 (Table 2). Interestingly, Zhu et al. (2022) performs best only on the topics “*Hillary Clinton”* and “*Donald Trump,”* while contrastive-learning-based approaches (Liang et al., 2022a,b) perform best on the other topics. Since Zhu et al. (2022) use external knowledge from Wikipedia, this likely benefits concrete topics (e.g., people) more than abstract topics (e.g., “feminism”), since articles on abstract topics typically place greater emphasis on historical and scholarly background. We note that although the cross-topic model from Liang et al. (2021), which incorporates pragmatic information, performs best on “*legalization of abortion,”* it is outperformed by zero-shot models on the remaining topics. Additionally, adversarial learning (Allaway et al., 2021) performs substantially better in the few-topic setting, compared to many-topic.

Overall, these results show that for zero-shot stance, general knowledge and features are more beneficial than modeling the latent space of topics or incorporating pragmatics. This aligns with intuitions about real-world scenarios where the number of topics is continuously growing. A fixed set of latent topics and training-set-derived pragmatics information are not well-suited to model an evolving discourse space. Instead, models that incorporate similarly evolving external knowledge or that can recognize general patterns of stancetaking (i.e., through general features) are more adaptable and better suited to such scenarios.

## 3. Extending zero-shot stance detection

We next survey existing work on multi-domain stance detection (Section 3.1) and then discuss important considerations for future studies on zero-shot multi-domain stance detection (Section 3.2).

### 3.1. Existing work

#### 3.1.1. Data

In multi-domain stance detection, the majority of work focuses on language as the domain (i.e., it is multilingual) (Taulé et al., 2017, 2018; Lai et al., 2020; Vamvas and Sennrich, 2020; Zotova et al., 2020; Hamdi et al., 2021). These datasets are primarily *not* intended for zero-shot scenarios, since the same languages and topics appear in both the training and evaluation sets. For example, multiple datasets have been created for stance detection on the topic of Catalonian independence with the Spanish and Catalan languages in both training and evaluation (Taulé et al., 2017, 2018; Zotova et al., 2020). Similarly, Lai et al. (2020) create a dataset for French and Italian with both the topics and languages shared across training and evaluation.

Additionally, there are a small number of datasets with subsets for ZSSD^*Topic*^ or ZSSD^*Language*^. Specifically, the *NewsEye* dataset (Hamdi et al., 2021) consists of historical news articles in four languages (German, French, Finnish, and Swedish) where, although all languages appear in the test set, the topics can be both zero-shot and not^2^. Going one step further, the *xstance* dataset (Vamvas and Sennrich, 2020) has both zero-shot topics *and* a zero-shot language (i.e., examples in Italian occur only in the test set). However, the zero-shot subsets of these datasets are still quite small. The *NewsEye* test subsets per language range from ~250 to ~1*k* instances. Similarly, in *xstance* there are only 1456 instances in the zero-shot language (i.e., Italian), of which only 283 also have zero-shot topics, compared to ~11*k* test instances with zero-shot topics in seen languages (German and French).

#### 3.1.2. Methods

The small amount of available data for multilingual settings has limited prior work. In particular, many works (e.g., Vamvas and Sennrich, 2020; Hamdi et al., 2021) focus on creating the dataset and provide only baseline results (e.g., from BERT Devlin et al., 2019). However, recent multi-domain studies that incorporate **multi-dataset learning** (Hardalov et al., 2021, 2022; Schiller et al., 2021) have increased the possibilities for multi-domain stance detection. In particular, multi-dataset learning makes available for training and evaluation a large number of stance datasets covering multiple languages, genres, and label sets.

Similar to techniques used for ZSSD^*Topic*^, work on multilingual and multi-genre stance also incorporates external knowledge, through task pre-training on GLUE (Schiller et al., 2021) or sentiment classification (Hardalov et al., 2022), as well as domain-invariant features. These domain-invariant features can be obtained through adversarial learning (Hardalov et al., 2021), as well as from label-embeddings, which allow multi-dataset learning with multiple label sets (Hardalov et al., 2021; Schiller et al., 2021). As with single-domain stance, external knowledge is particularly effective for multi-domain stance in both multilingual and multi-genre settings.

#### 3.1.3. Evaluation and difficulties

Despite these techniques, there still remain large performance gaps between fully-supervised and zero-shot models. For example, although Hardalov et al. (2021) evaluate on 16 English datasets (from multiple genres), only VAST is for ZSSD^*Topic*^, and Hardalov et al. (2021)'s best model is 49.3% below SOTA (i.e., Liu Y. et al., 2022). Similarly, in a multilingual setting, the performance drop for zero-shot evaluation is 39.4% on average (across 15 datasets from 12 languages), dropping below random guessing for 1/3 of the datasets (Hardalov et al., 2022). Furthermore, for 60% of the datasets, the best model is trained on stance datasets only in English. Note that in Hardalov et al. (2022), zero-shot refers to whether a particular *dataset* is seen during training and this is problematic.

In fact, vagueness surrounding the notion of ZSSD makes the results of prior work difficult to evaluate clearly. In particular, the multilingual zero-shot experiments of Hardalov et al. (2022) cannot be considered ZSSD^*Language*^ because languages occur in multiple datasets (e.g., Italian and French both occur in two). The experiments are similarly not ZSSD^*Genre*^ because multiple datasets from a single genre are used (e.g., seven Twitter datasets are used). Finally, these experiments are not ZSSD^*Topic*^ because, although, many datasets contain a unique single topic (e.g., “Emmanuel Macron”) topic overlap does exist (e.g., in *xstance*) and may be exacerbated by pre-training on English stance datasets (i.e., in the best model for many datasets).

Work on multi-genre stance in English from Hardalov et al. (2021) exhibits similar issues. Specifically, the out-of-domain experiments cannot be considered ZSSD^*Genre*^ because multiple other datasets from the same genre as the test dataset are used for training (e.g., Twitter) Furthermore, these experiments are also not ZSSD^*Topic*^ because multiple datasets share topics (e.g., “Donald Trump” in Mohammad et al., 2016; Sobhani et al., 2017).

Therefore, despite promising recent work on multi-domain stance detection, there is significant room for improvement in zero-shot settings, in terms of both empirical results and controlled evaluation settings.

### 3.2. Proposed best practices

In order to support future work on ZSSD and address the evaluation problems of prior work, we propose best practices for ZSSD training and evaluation. First, in order to conduct such experiments it is important to **specify zero-shot development sets** (i.e., for hyperparameter tuning). For example, in ZSSD^*Language*^, at least one language that is *different* from the training and test sets should be designated for development. This ensures the test set remains zero-shot while providing an approximation of zero-shot performance for tuning.

Secondly, domain aspects intended for zero-shot evaluation should be **explicitly designated** and **overlap controlled for** within these aspects (i.e., between training and evaluation). This is especially important in multi-dataset learning, since multiple datasets may share the value of a domain aspect (e.g., have the same language). Controlling for overlap ensures that experiments accurately measure ZSSD.

Finally, for the set *A* of zero-shot domain aspects being studied (e.g., zero-shot languages and topics) **all combinations of the aspects should be evaluated**. That is, experiments should be conducted on ZSSD^α^ for every non-empty α⊆*A*, in order to explicitly distinguish improvement in particular types of zero-shot transfer. For example, if *A* = {*Topic, Language*}, then we should evaluate not only on ZSSD^*Topic, Language*^, but also ZSSD^*Topic*^ and ZSSD ^*Language*^.

We hope that these propositions will support and systematize research in ZSSD.

## 4. Discussion

Regardless of the setting, ZSSD presents a number of ongoing challenges: evaluating for **robustness** and **explainability**. Although these are not unique to ZSSD, they are important considerations for ZSSD due to the sensitive nature of many stance topics (e.g., political or ideological beliefs).

Robustness is important because overall empirical improvements can be misleading. Consider the VAST dataset, which was constructed with a designated challenge component to probe complex language (e.g., sarcasm) and potentially spurious signals in the data (e.g., examples that share a document but have different topics). Only two systems report results for this set (i.e., Liu et al., 2021; Liu R. et al., 2022) and the performance drops across phenomena range from 0.1 to 21.5%. Although, Liu et al. (2021) has higher zero-shot *F*1 (see Table 2), it has larger performance drops on 4/5 types of challenging phenomena. Therefore, considering only overall *F*1 is problematic for evaluating models.

Adversarial attacks can also be used to probe robustness. For example, we (Allaway and McKeown, 2020) find performance differences ranging from 1.3 to 9.5% due to changes in sentiment of an input. Alternatively, Schiller et al. (2021) conduct adversarial attacks that introduce paraphrasing, spelling errors, and unnecessary negation. They find that although multi-dataset learning outperforms single-dataset learning across datasets, the multi-dataset model is twice as susceptible to adversarial attacks (i.e., an average drop of 10.3% for multi-dataset learning, compared to 5.7% for single-dataset learning). This further illustrates the necessity of comprehensive evaluation for models.

Explainability is especially important in ZSSD, where predictions on zero-shot instances should be grounded in valid human reasoning. Although, we (Jayaram and Allaway, 2021) have investigated training a model's attention to mimic human rationales, there has been limited work on explainability for stance detection. Since models incorporating external knowledge exhibit particularly strong performance for ZSSD^*Topic*^ (see Section 2.3), explainability can help to validate that model predictions are based on reasonable and true facts or inferences. This will in turn increase both human confidence in the models and real-world viability.

In this work, we present two paradigms for ZSSD^*Topic*^ (i.e., zero-shot topics) and review existing methods and data for each. We argue that incorporating external knowledge and domain-invariant features are the most promising techniques. Additionally, we argue that ZSSD should be expanded beyond English and we analyze work in multi-lingual and multi-genre stance (including non-ZSSD). Although multi-dataset learning is promising, there is still significant room for improvement, particularly in zero-shot settings. To stimulate further work and systematize evaluation, we propose a set of best practices for ZSSD. Finally, we argue that robustness and explainability should be considered both in the construction and evaluation of ZSSD models.

## Data availability statement

Publicly available datasets were analyzed in this study. This data can be found at: https://github.com/emilyallaway/zero-shot-stance/tree/master/data/VAST; https://github.com/MalavikaSrikanth16/adversarial-learning-for-stance/blob/main/src/data/twitter_data_naacl.zip.

## Author contributions

EA: writing, analysis of the datasets, methods, and discussion. KM: advising and editing. All authors contributed to the article and approved the submitted version.

## Footnotes

^1^Also called “cross-target” (e.g., in Mohammad et al., 2016; Xu et al., 2018).

^2^instances are not labeled explicitly as having zero-shot topics or not.
